# Genetic recombination in plant-infecting messenger-sense RNA viruses: overview and research perspectives

**DOI:** 10.3389/fpls.2013.00068

**Published:** 2013-03-26

**Authors:** Jozef J. Bujarski

**Affiliations:** ^1^Plant Molecular Biology Center and the Department of Biological Sciences, Northern Illinois UniversityDeKalb, IL, USA; ^2^Laboratory of Molecular and Systems Biology, Institute of Bioorganic Chemistry, Polish Academy of SciencesPoznan, Poland

**Keywords:** RNA recombination, viral replicase, template switching, non-replicative RNAs, host factors, cellular RNAs, ribonucleases, viral evolution

## Abstract

RNA recombination is one of the driving forces of genetic variability in (+)-strand RNA viruses. Various types of RNA–RNA crossovers were described including crosses between the same or different viral RNAs or between viral and cellular RNAs. Likewise, a variety of molecular mechanisms are known to support RNA recombination, such as replicative events (based on internal or end-to-end replicase switchings) along with non-replicative joining among RNA fragments of viral and/or cellular origin. Such mechanisms as RNA decay or RNA interference are responsible for RNA fragmentation and *trans*-esterification reactions which are likely accountable for ligation of RNA fragments. Numerous host factors were found to affect the profiles of viral RNA recombinants and significant differences in recombination frequency were observed among various RNA viruses. Comparative analyses of viral sequences allowed for the development of evolutionary models in order to explain adaptive phenotypic changes and co-evolving sites. Many questions remain to be answered by forthcoming RNA recombination research. (1) How various factors modulate the ability of viral replicase to switch templates, (2) What is the intracellular location of RNA–RNA template switchings, (3) Mechanisms and factors responsible for non-replicative RNA recombination, (4) Mechanisms of integration of RNA viral sequences with cellular genomic DNA, and (5) What is the role of RNA splicing and ribozyme activity. From an evolutionary stand point, it is not known how RNA viruses parasitize new host species *via* recombination, nor is it obvious what the contribution of RNA recombination is among other RNA modification pathways. We do not understand why the frequency of RNA recombination varies so much among RNA viruses and the status of RNA recombination as a form of sex is not well documented.

## Introduction

Plus-stranded RNA viruses include some of the most dangerous pathogens for animals and humans. Moreover, a vast majority of plant viruses are (+) RNA viruses. RNA viruses demonstrate a large level of variability in their genetic information, due to either mutations, RNA–RNA crossovers (RNA recombination), or reassortment. RNA recombination was demonstrated for many RNA virus species, whether under natural or experimental conditions. Similar to genetic recombination in DNA-based organisms, viral RNA recombination is defined as the process of swapping RNA fragments among RNA molecules. If crossovers occur amongst the same RNA templates in a homologous fashion, the exchanges are functionally equivalent to DNA meiotic crossing-over. In some viruses, the frequency of homologous crossing-over is very high and practically every replicated viral RNA molecule can be considered as chimerical in nature, as we have demonstrated for brome mosaic virus (BMV) RNAs (Urbanowicz et al., 2005).

**Figure 1:**
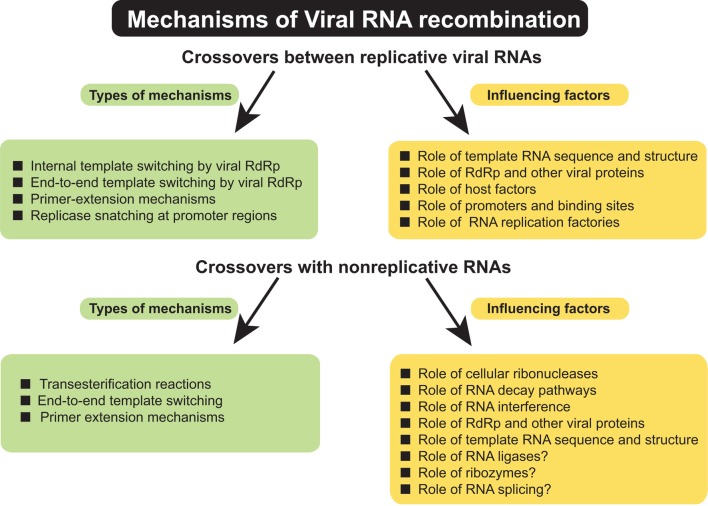
**Summary of types of mechanisms of genetic RNA recombination in (+) RNA viruses and factors influencing the RNA–RNA crossovers**.

A variety of events have been described that contribute to the formation of RNA recombinants (Figure 1). Such events include crossovers between viruses belonging to the same or to different taxonomic groups, between viruses infecting different hosts, or from adopting genetic material from the host. Numerous questions about molecular mechanisms of RNA recombination remain unanswered. This review attempts to summarize the most important venues of RNA recombination research, their challenges and future directions in order to draw more accurate models for this important RNA virus phenomenon. Since this issue of Frontiers concerns plant pathology, most of the material discusses RNA recombination in plant viruses. However, the less advanced aspects of plant recombination studies have been illustrated with examples taken from animal/human RNA viruses in order to show mutual possibilities for model research.

## Replicative mechanism of RNA recombination

The generally accepted mechanism of RNA recombination is currently explained by a copy-choice model where the viral RNA polymerase (RdRp) complex in mRNA viruses [reverse transcriptase (RT) in retroviruses] changes templates during synthesis of the nascent strand (Galetto et al., 2006). This swapping process generates recombinant RNA molecules of mixed ancestry. Although we begin to understand the nature of these processes, many questions are waiting for an answer. One group of questions revolves around the features that define the sites of crossovers. Among the factors known to promote replicase to switch are sequence homologies between recombination substrates along with secondary structures at the crossover sites, as demonstrated with the BMV and other systems (Figlerowicz and Bujarski, 1998; Nagy et al., 1999b). Also, the transcriptional activity seems to promote template switching. For instance, an efficient recombination hot spot has been mapped within the intercistronic region of BMV RNA3, the site carrying the promoter of transcription of subgenomic RNA4 (Wierzchoslawski et al., 2004). It is unknown what exactly facilitates crossovers at such sites. Possibilities include a snatching process of already bound RdRp complex to the promoter site, the premature termination of RNA synthesis and the replicase detachment–reattachment, or the effect of other bound viral and/or host factors (Sztuba-Solinska et al., 2011). These mechanisms may depend upon the type of template-switching process (whether the crosses occur internally or near the ends of the RNA templates) and on the involvement of crossover sequences in other processes, e.g., as a promoter of RNA replication or transcription.

Template switching was found to occur between related but also between unrelated RNA templates, generating legitimate (homologous) and illegitimate (nonhomologous) recombinants, respectively (Nagy and Simon, 1997). Since the latter involves sequences with little similarity, other factors must be important. Some data indicate that switches depend upon sequence composition, with the AU-rich regions promoting the RdRp detachment (Nagy et al., 1999a) and upon secondary structures (Galetto et al., 2006), along with protein or RNA binding activity. Switching may also depend upon the processivity (a measure of the average number of nucleotides copied per template association–disassociation cycle) features of the RdRp enzyme (Breyer and Matthews, 2001). A mandatory replicase breaking site is the end of any RNA template. End-to-end switching has been reported based upon *in vitro* results with RdRp enzymes from Bovine viral diarrhea virus (BVDV), BMV, Cucumber mosaic virus (CMV), and Cowpea chlorotic mottle virus (CCMV) (Kim and Kao, 2001). It is, however, not known how exactly such switches occur and whether the molecular mechanism is common among polymerases of different RNA viruses.

The strength of binding of the RdRp complex may play a key role during RNA template detachment–reattachment. With an increasing number of available RNA polymerase crystal structures, more is evident about the elements involved in RNA-replicase interactions. For instance, removal of a β-hairpin loop from the HCV RdRp protein increased *de novo* RNA synthesis and promoted RNA binding (Mosley et al., 2012). The RNA copying fidelity might be a matter of a nanosecond timescale complex dynamic in the RdRp enzyme that determines RNA binding, nucleotide binding or catalysis (Moustafa et al., 2011) and thus needs to be experimentally determined. The use of engineered replicase variants in RNA recombination assays will shed new light onto the molecular details of template switching mechanisms.

Another, not well answered question is how RNA template substrates come together in order to facilitate the switch. One possibility is that secondary structure regions can hybridize in *trans* bringing the two RNA templates into a local interaction. Such data are available, for instance, based upon limited observations in BMV (Nagy and Bujarski, 1993; Dzianott et al., 1995) or analogously, during switches between dimeric RNAs (within kissing loops) during reverse transcription inside the Human immunodeficiency virus Type-1 (HIV-1) virions (Nikolaitchik et al., 2011), an atypical (+) sense RNA virus.

Yet, other data reveal that (+) RNA viruses are replicating in membranous structures called spherules or replication factories (Laliberté and Sanfaçon, 2010). Such host membrane-derived replication vesicles have limited loading capacity, but they may carry up to several positive and negative strand RNA molecules (den Boon et al., 2010). Recent advances reveal the assembly of replicase complexes within replication factories *via* highly orchestrated interactions between viral proteins, viral genomic RNAs, and co-opted host factors (Mine and Okuno, 2012). Such a micro-environment may secure tight packaging and thus the closeness of internalized viral RNA molecules. From the formal stand point then, one may consider RNA recombination switches in (+) RNA viruses inside replication factories as analogous to the switches that occur, e.g., during reverse transcription inside the HIV-1 virions.

Recently, we have demonstrated the participation of coat protein (CP) during BMV RNA recombination (Sztuba-Solinska et al., 2012). The nucleotide changes in *cis*-acting RNA motifs and the amino acid replacements within the corresponding CP binding sites—both debilitated the BMV RNA recombination. CP molecules likely mediate RNA crosses *via* dimerization/oligomerization of bound CP subunits. Indeed, the presence of BMV CP molecules has been demonstrated to be inside replication vesicles (Bamunusinghe et al., 2011). Another untested possibility predicts that a bound CP functions as a road block catalyzing the detachment of the replicase complex. The CP may also affect the properties of viral replicase. For instance, it has been shown recently that Norovirus RNA synthesis was enhanced by co-expressed structural protein VP1 (Subba-Reddy et al., 2012).

## Recombination with non-replicative RNAs

Besides replicative copy-choice, the non-replicative mechanisms of viral RNA recombination have been described, mainly for animal/human RNA viruses, with almost no research focusing plant viruses. One of the best characterized non-replicative processes is demonstrated in the poliovirus where viable viruses were rescued in cells co-transfected with different pairs of viral RNA fragments (Gmyl et al., 1999). It is likely the recombinants may have resulted from transesterification reactions with the end structures similar to known ribozymes *via* intermediary formation of 2′,3′-cyclic phosphate. Indeed, *in vitro* data show that the transesterification reactions in the bacteriophage Qbeta RNA are guided by secondary structures that direct the attack of a 3′ hydroxyl onto the phosphodiester bonds (Chetverin et al., 1997). Later observations revealed enormous variability of the poliovirus genome and some variants may have been introduced by genetic errors due to non-replicative mechanisms (Agol, 2006). More recent results with partially-complementary RNA-oligonucleotides demonstrated the spontaneous formation of novel RNA molecules *via* 3′,5′-phosphodiester bonds (Lutay et al., 2007). These data show that viral RNA recombination can occur without participation of the RNA polymerase enzyme. The exact mechanisms of these non-replicative events are not completely understood and require further studies.

In contrast to poliovirus and other picornaviruses, bacteriophage Qbeta demonstrates low levels of recombination frequency. By using a cell-free system, Chetverin et al. (2005) have shown a high yield of primer-extension recombination with poliovirus replicase, but a low yield with Qbeta replicase. Thus, RNA recombination by poliovirus *vs*. Qbeta RdRps must be mechanistically different. Although both utilize transesterification reactions, the precise molecular bases for RNA swappings used by each of these enzymes are likely dissimilar reflecting different biochemical adaptations to the needs of individual viruses. It would be interesting to confirm experimentally the proposed transesterification models.

Among other examples of non-replicative recombination in mRNA viruses, the co-transfections of replicating and nonreplicating rubella virus (RUB) RNA transcripts containing nonoverlapping deletions did restore the infectious virus (Adams et al., 2003). Both, homologous and nonhomologous RNA recombinants emerged. The mechanism seemed to involve end-to-end replicase switching after initiation of minus-strand synthesis. However, the details of such mechanisms have not yet been confirmed. Another example of that sort involves recombination between BVDV and cellular RNAs, which can occur in the absence of viral replicase (see section “Recombination Between Viral and Cellular RNAs”). Analogous studies in the area of plant virology remain to be performed.

## Role of host factors during RNA recombination

An important subject of RNA recombination research is the role of host factors. While the involvement of viral RdRp proteins has been studied extensively, knowledge of the functions host components play is limited (Nagy and Pogany, 2011). One study was done with a model system of tomato bushy stunt virus (TBSV) that can recombine in yeast cells. The authors screened a yeast knockout library to identify over thirty different host genes suppressing or accelerating the TBSV RNA recombination (Serviene et al., 2005; Nagy, 2011). An interesting example is the gene PMR1 which encodes an ion pump (Pmr1p) controlling the Mn2+ concentration which may consequently affect the ability of TBSV replicase to change RNA binding/template switching events. Also stress signals, e.g., salt stress, affect viral recombination indirectly, by changing the concentration of recombination-essential proteins. Future studies are required to understand the interrelated network of cellular factors that define the final outcome of TBSV RNA recombinants, not only in model yeast cells, but also in natural TBSV plant hosts. Moreover, these studies are limited to only one specific TBSV RNA experimental recombination system, and it is unclear if other RNA recombination events within the TBSV RNA follow similar mechanistic pathways.

In the copy choice mechanism, recombinant RNAs are formed due to switching of viral replicase among RNA templates. The switching properties likely depend on the co-recruited host factors. In BMV, a variety of host factors were found to be employed by the replicase complex (Noueiry and Ahlquist, 2003). Many of these factors facilitate the complex assembly, but some regulate viral gene expression or recruitment of BMV RNAs to the membrane replication factories. Yet, other factors modify lipid composition of the endoplasmic reticulum membrane which activates the replication complex. Many of these factors can potentially affect the co-recruitment of RNA recombination substrates and/or BMV replicase switching properties during recombination. BMV RNA recombination was reported to occur in yeast cells (Garcia-Ruiz and Ahlquist, 2006), but a systematic identification of host factors participating in BMV RNA recombination remains to be done. It will be interesting to find out whether these factors parallel those in the above tombusvirus recombination system. This data will broaden our knowledge about host pathways enabling RNA viruses to recombine their genetic information. As such, it will contribute to predictions made on the stability of the RNA viral genome in various hosts.

The functions of recruited host proteins and host membranes in different (+) RNA virus systems are now being progressively elucidated. Comparison among three plant RNA virus replication systems (TBSV, BMV, and dianthoviruses) reveals general patterns within the stepwise process of viral replicase complex assembly which requires concerted involvement of protein–protein, RNA–protein, and protein–lipid interactions (Mine and Okuno, 2012). However, each of these three plant virus systems recruits its own array of specific host factors. This suggests that each RNA virus has significantly unique ways of adapting to the cellular environment in order to assemble a functional RNA replication complex. This further suggests specific requirements are needed for RNA recombination in each individual RNA virus and therefore the recombination characteristics may significantly differ with each other among RNA viruses. Crystal structure studies help to reveal the complex and individual nature of viral replicases. Examples being the structure of Q{beta} phage polymerase, determined by Takeshita and Tomita (2010), or the analysis of the crystal structure of tomato mosaic virus helicase as a component of the viral replicase complex (Nishikiori et al., 2012).

## Recombination between viral and cellular RNAs

RNA recombination events between viral and cellular RNAs have been observed for both plant and animal RNA viruses. One example is RNA recombination between the BVDV, a member of the pestivirus genus, and cellular RNA sequences. It occurs at the presence yet also in the absence of an active viral RdRp enzyme, implying that the mechanisms must be different from replicative template switching events (Becher and Tautz, 2011). The case of BVDV recombination has practical implications because the recombinant virus is lethal to its host. Normally, the virus is persistent, limiting the efficiency of RNA replication due to the dependency of a viral protease on limiting amounts of a cellular cofactor. In general, the uptake of a variety of cellular protein coding sequences at various positions in the pestiviral genomes has been reported, demonstrating that pestiviruses can gain access to the RNA pool of their hosts *via* RNA recombination. The example of BVDV shows us not only that recombination events with cellular RNAs cannot be excluded for other viruses, but also that the recombinant RNAs can be retro-transcribed and occasionally integrated into the host genome. The exact molecular mechanisms of the crossover events with cellular RNAs remain to be elucidated, as well as what factors target the crossover sites both to viral and to cellular RNAs.

Besides BVDV, HIV-1 is known to recombine effectively with host RNAs, e.g., with host tRNAs after introducing its strong secondary structure elements into the HIV RNA (Konstantinova et al., 2007). HIV-1 is capable of acquiring new genetic material, especially to the RT-encoding ORF (van der Hoek et al., 2005; Berkhout, 2011). Information about similar recombinant crosses with host RNAs in plant RNA viruses remains very limited, and their mechanisms are waiting to be elucidated.

One recombination process that was addressed with plant viruses has been the events between an invading virus and the transgene mRNAs in transgenic plants (Aaziz and Tepfer, 1999). One such example being recombination between two strains of CMV where one strain was expressed as a transgene while the other strain infected the transgenic plant (Turturo et al., 2008). This research group has also described recombination between related viruses (CMV and tomato aspermy virus TAV), with the population of recombinants being similar to each other in transgenic and in nontransgenic plants, suggesting similar molecular mechanisms of recombination (Jacquemond, 2012). In general, this demonstrates that transgene viral mRNAs enter the same pathway as do natural viral RNAs, most likely operating in the cytoplasm.

RNA recombination between viral and micro (mi)RNAs has not yet been reported. However, given the fact that this would be a useful source of already adapted elements to be acquired by the virus in order to secure the in-*trans* host-gene regulation, the lack of commonality of such an acquisition is surprising. Since (+) RNA viruses operate in the cytoplasm, as the miRNAs do, there are likely either structural and/or functional constrains against such snatching events. Future studies will certainly bring further insight to this question.

Recently, a reverse scenario was observed. Nonretroviral RNA sequences of Bornaviruses and other (−) strand RNA viruses were integrated into the host genome, including the human genome (Belyi et al., 2010; Horie et al., 2010). Also, mRNA viruses were described to leave their sequences in the cellular DNA of infected hosts (Crochu et al., 2004; Anne and Sela, 2005; Maori et al., 2007; Zemer et al., 2008; Geuking et al., 2009). These results demonstrate that RNA viruses can serve as a source of genetic innovation for their hosts. The RT activity encoded by retrotransposons is most likely responsible for reverse transcription and integration, yet further molecular studies are needed.

The above examples illustrate that the cytoplasmic RNA processing mechanisms are able to cross paths with viral replication pathways inside the cell. Despite diverse examples of viral RNAs recombining with host RNA sequences (and *vice versa*), many unanswered questions remain to be addressed. They include, but are not limited to, the sub-cellular location of recombination events, the role and availability of host RNA degradome for recombination, or the link between the elements of RNA degradation pathways and viral RNA recombination. The molecular mechanisms of such crossover events are not well understood, especially whether template-switching or re-ligation processes are involved. More data, especially from plant RNA virus systems are required to assess the general nature of these processes in plant vs. animal/human tissues.

## Role of ribonucleases and RNA interference pathways

Host RNAs undergo extensive degradation and turnover, as do viral RNAs (Lloyd, 2012). The participation of RNA decay pathways in viral RNA recombination has been studied in TBSV by the Nagy group (Jiang et al., 2010; Jaag et al., 2011). By testing eight known endoribonucleases, the authors have shown that mutations in the components of RNase MRP debilitated the production of endoribonucleolytically cleaved TBSV RNA in yeast. Also, by knocking down the RNase NME1 or silencing the Xrn4p exoribonuclease in *Nicotiana benthamiana*, the production of cleaved TBSV RNAs was debilitated, but recombination increased, suggesting the role of RNA intermediates in recombination (Jaag and Nagy, 2009). Similar effects promoting RNA recombination were observed in yeast for Xrn1p exoribonuclease (Serviene et al., 2005). It is noteworthy that deletions of the host Met22p/Hal2p bisphosphate-3′-nucleotidase (a known inhibitor of the Xmn1p ribonuclease) or the inhibition of this nucleotidase with LiCl or NaCl, also increased the frequency of TBSV RNA recombination in yeast (Jaag and Nagy, 2010). This shows that besides host factors, the salt stress can also affect viral RNA recombination. Whether other environmental conditions can influence viral RNA recombination needs further studies.

In contrary to RNA decay enzymes, we observed debilitating effects of the host RNA interference gene knockouts on BMV RNA recombination in *Arabidopsis thaliana*, and that BMV RNA fragments have recombined with BMV RNA progeny (Dzianott et al., 2012). It appeared that RNA silencing (RNAi) pathways participated in the rearrangement of genomic BMV RNAs. Therefore, BMV RNAs can recombine *via* several mechanisms including template-switching events along with RNAi-based sequence swapping. Similarly, the promoting role of RNAi in viral RNA recombination was reported for mycovirus infection in chestnut blight fungus cells (Sun et al., 2009; Nuss, 2011). These two examples show that the RNAi mechanisms can function as antiviral tools, but also that RNA silencing can promote additional variability to the viral RNA genome. Further studies are needed to determine the formation of viral RNA recombinants from RNAi-induced degradation products.

## The phylogenetic and evolutionary role of RNA recombination

The biological diversity within both plant and animal RNA viruses is one of the largest found in all other forms of nature. RNA recombination is a main contributor to the ever evolving RNA viral genome. Comparative analyses of RNA viral sequences allow for the development of evolutionary models that demonstrate the associated adaptive phenotypic changes along with detecting the co-evolving sites within viral genomes (Pond et al., 2012).

The wide imprints of RNA recombination were found within natural populations of plant viruses. RNA recombination seems to be particularly frequent among members of the family *Potyviridae*, the largest family of plant RNA viruses. Frequent recombinational footprints were detected within the ORFs of both their structural and nonstructural proteins (Bousalem et al., 2000; Visser and Bellstedt, 2009; Yamasaki et al., 2010). Phylogenetic surveys indicate not only intraspecies and intragenus, but also intergenous recombination crossover's footprints in *Potyviridae* (Desbiez and Lecoq, 2004; Valli et al., 2007), supporting their apparent modular evolution. Recombination with host RNA was also detected, likely via retrotransposable elements (Tanne and Sela, 2005) demonstrating that, like animal viruses, plant viruses can expand their coding capacity via recombination with the host's messenger RNA pool (Chare and Holmes, 2006).

Also, the populations of plant viruses with genomes producing sgRNAs, e.g., *Closteroviridae*, *Luteoviridae*, or viruses with multipartite genomes, e.g., *Bromoviridae*, seem to accumulate recombinants readily. Evolutionary pathways were proposed for the emergence of members of *Luteoviridae* (Domier et al., 2002 and Moreno et al., 2004). Luteoviruses have mastered the process of modular swap (Pagán and Holmes, 2010) and the reconstructed phylogeny reveals their sequence evolution by intrafamilial as well as extrafamilial RNA recombination (Moonan et al., 2000). The most frequent swaps map to the junction between the CP and RdRp ORFs (Silva et al., 2008). In addition, some luteoviruses were found to recombine with host (chloroplast) sequences (Mayo and Jolly, 1991).

One extreme example of interspecies recombination is in circoviruses that arose by recombinants between plant DNA nanoviruses and mammalian RNA caliciviruses. In this case, the likely mediator has been a retrovirus that retro-transcribed the RNA into DNA (Davidson and Silva, 2008). Although likely, such events have not been experimentally confirmed and further research is required.

Among animal viruses, coronaviruses are highly recombinogenic (Woo et al., 2009) and natural RNA recombinant variants were described for flaviviruses (González-Candelas et al., 2011). By having one of the highest recombination rates among all viruses, retroviruses generate polymorphic sequences that increase their chances for survival under changing selection pressures (Delviks-Frankenberry et al., 2011). Besides retroviruses, picornaviruses are naturally highly recombinogenic (Lukashev, 2010). In fact, RNA recombination is their key genetic feature maintaining the global pool of variants from which the recombination snapshots generate new recombinant forms of picornaviruses. For instance, a model describing recombination between poliovirus and coxsackie virus was presented to illustrate the effects on viral emergence and evolution (Combelas et al., 2011). This and other studies reveal multiple mechanisms leading to genetic variability of polioviruses (Savolainen-Kopra and Blomqvist, 2010), with significant contribution of homologous recombination events that fix advantageous mutations or remove deleterious ones. However, further research is required to understand the detailed evolution mechanisms of polioviruses.

The evolutionary genetics of emerging plant RNA viruses was studied by Elena et al. (2011). Apparently, devastating virus epidemics can spread from new plant virus variants that acquired new virulence factors. This study shows a multifaceted picture of virus emergence. Changes in ecological conditions bring together the reservoir viruses and their crop hosts, often as a result of interplay among the environment, genetic plasticity, and the required host factors. The stochastic processes contribute to the beginning of viral emergence in a new host species, followed by the adaptation phase. Also, vectors impose strong bottlenecks during host-to-host transmissions. The reservoir population seems to be the most important determinant of viral emergence, but little is known about viruses of wild plant species that work as reservoirs.

For all the mentioned RNA virus systems, of either plants or animals, detailed roles during virus evolution of RNA secondary structures, the function of sequence similarity or the impact of RNA co-packaging during RNA recombination, are all not well understood. Inaccuracies of viral RNA replication, damage from environmental factors, and attacks by RNA-modifying enzymes, all can contribute to RNA genome corruption and thus generate a question of how RNA viruses maintain their genetic integrity (Barr and Fearns, 2010). It seems that viral RdRps are sufficiently flexible to accommodate alternative initiation mechanisms, enabling terminal repair, terminal transferase activity, and recombinational crosses in the case of damaged key terminal sequences. Among a variety of mechanisms to protect RNA viral genome integrity, recombination allows for exchange of sequences between RNA templates, protecting not only their entire genome, but also their vulnerable termini. A typical example of efficient terminal crossover exchanges is seen within the 3′ noncoding region of BMV RNAs (Bujarski and Kaesberg, 1986). The differences in replicase architecture might affect the predilection of a particular virus for RNA recombination. The molecular aspects of the theory on “adaptable” viral RdRps have not been elucidated and structural studies will contribute to the answers.

## Methods for the detection of RNA recombinants

RNA recombination research concerns both virus evolution (where the most important subject is the detection of recombination imprints among natural viral RNA sequences) and the mechanism of recombination (by using the experimental systems of enhanced recombination frequency). As regards to the evolutionary studies, various computer programs are used for massive comparisons of viral sequences in order to reveal the recombination footprints. The examples of such software include, but are not limited to, Topali, RECCO, GARD, RDP, GENECONV, Chimaera, MaxChi, BOOTSCAN, SISCAN, PHYLPRO, DIPLOMO, SImPlot, Lard, and 3SEQ. These programs can identify the recombination sites among different viral strains, different viral species, and even between the virus and the host (Chare and Holmes, 2006). With advent of next-generation massive sequencing, the genetic diversity of viral RNA genomes can be characterized through the mapping of polymorphisms and measurement of mutation frequencies as well as by detection of recombination events to a single-nucleotide resolution (Routh et al., 2012). Such approach is very sensitive and unbiased, and it can identify hundreds of thousands of recombination events, allowing for a detailed description of RNA crossover profiles.

The detection of recombination events in the laboratory is challenging because RNA–RNA crossovers apparently are rare events and thus the main effort is to elaborate on experimental systems of engineered RNA templates of increased recombination activity. The efficient recovery of recombinants in mixed infections could be achieved by using temperature sensitive mutants, a long-term method used for animal RNA viruses (Hirst, 1962; Pringle, 1970). In whole plants, an important problem is that most recombinants are not competitive with the parental types and therefore disappear. One way to increase recombination rate is by using viral mutants bearing sequence modifications at their UTRs, which decreases the replication abilities of parental molecules, as was used to detect the BMV recombinants (Bujarski and Kaesberg, 1986). Another approach utilizes viral RNAs bearing silent markers or via mixed infections with two low-competing viral strains (e.g., as the used by us mixed infection with both type and Fescue strains of BMV). Other plant virus recombination systems employ mixtures of two parental RNAs with one component carrying a deleterious mutation, e.g., satellite and genomic RNAs of TCV (Zhang et al., 1991), or defective interfering and genomic RNAs of TBSV, Cucumber necrosis virus (CNV) (White and Morris, 1995), and Potato virus X (PVX) (Draghici and Varrelmann, 2010). All these types of recombination systems can be used in cell-free extracts (utilizing viral RdRp preparations), in single-cell (protoplast) hosts, in whole plant hosts, and even in yeast. Some of the systems make use of transient expression vectors by agro-infiltrating plant leaves (Kwon and Rao, 2012).

With these systems in hand, virologists can address such aspects of the RNA recombination process as the essential role of RNA sequence and structure, especially the role of RNA motifs, the function of viral replicase (RdRp) and other viral- and/or host-encoded proteins, or the mutual host–virus effects in short-term virus evolution. The main analytical effort in the recombination experiments is to identify RNA recombination products and to map the location of cross sites. Usually, viral RNAs are extracted and amplified by RT-PCR and the resulting cDNA products are cloned followed by sequencing and/or restriction digestion of a large number of clones. This way the crossovers are detected and mapped within the sequence markers, providing information about both frequency and distribution of recombination events. Proper controls are required to guard against RT-PCR generated recombinants.

## Final remarks: unanswered questions and perspectives

Genetic RNA recombination is a major driving force for RNA virus diversity. By understanding the factors and the mechanisms that affect recombination, one can ultimately develop better means for controlling RNA virus infections. In this review I have described the current status of RNA virus recombination research and its future directions. I have also noted its progress over the last several years emphasizing on some future research venues. Evidently, there is still much to be learned about the mechanistic details of RNA recombination. For example, it is not yet clear how various factors modulate the ability of viral replicase to switch templates, such as the role of RNA template structures, the molecular and structural features of replicase proteins, or the functions of other viral and host factors during cross-over events. Also, the intracellular location of the RNA–RNA template switching has not been confirmed. Besides copy-choice, RNA viruses can recombine with non-replicative RNAs. It is not exactly known what mechanism is responsible for ligation of viral RNA fragments, or where inside the cell this process occurs. RNA viruses were found to recombine with cellular RNAs, but again where in the cell and what factors enable such events, is not well known. And the opposite, the exact steps and the molecular mechanisms of the RNA viral sequence integration with the cellular DNA have not been untangled. Amongst other questions, not much is known about how splicing or active ribozymes can contribute to the RNA virus recombination (Edgell et al., 2011).

From the evolutionary stand point, RNA recombination seems to play a key function during virus speciation and emergence, but its shared contribution that parallels other RNA modification pathways has not yet been assessed. We do not fully understand how RNA viruses achieve their high potential of parasitizing new host species *via* recombination (Domingo, 2010). The entire population of RNA variants that are present in reservoir hosts can now be determined with the tools of next-generation sequencing so that the role of recombinants can be more precisely evaluated (Beerenwinkel et al., 2012).

The frequency of RNA crossing-over varies among RNA virus species and there is little evidence that recombination was favored by natural selection. Because of this and since recombination rates follow the patterns of RNA genome organization, Simon-Loriere and Holmes (2011) postulate that RNA recombination is a by-product of viral genome arrangement acting on selected aspects of the virus life cycle. Thus, according to the authors, RNA recombination does not seem to function as an obligatory form of sex in RNA viruses. Yet further studies are required, especially since Muller's ratchet effects were observed in RNA viruses (Turner, 2003) and the chimeric nature of viral RNAs due to frequent homologous RNA swaps was determined, e.g., in BMV (Urbanowicz et al., 2005).

Despite the above deficiencies, the so far accumulated knowledge about viral RNA recombination has already found some practical applications. For example, measures could be taken to reduce recombination while designing the antiviral resistance in transgenic plants with artificial micro RNAs (Fahim and Larkin, 2013) or with double stranded RNA-expressing transgenes (Zhang et al., 2011). Also, the potential instability and recovery of the wild-type virus *via* recombination can be reduced during construction of plant RNA viral vectors (Nagyová and Subr, 2007).

### Conflict of interest statement

The author declares that the research was conducted in the absence of any commercial or financial relationships that could be construed as a potential conflict of interest.
